# High Silica Content Graphene/Natural Rubber Composites Prepared by a Wet Compounding and Latex Mixing Process

**DOI:** 10.3390/polym12112549

**Published:** 2020-10-30

**Authors:** Jian Wang, Kaiye Zhang, Guoxia Fei, Martina Salzano de Luna, Marino Lavorgna, Hesheng Xia

**Affiliations:** 1 State Key Lab of Polymer Materials Engineering, Polymer Research Institute, Sichuan University, Chengdu 610065, China; wangjian@cdu.edu.cn (J.W.); 18354212670@163.com (K.Z.); xiahs@scu.edu.cn (H.X.); 2 College of Food and Biological Engineering, Chengdu University, Chengdu 610065, China; 3 Institute of Polymers, Composites and Biomaterials, National Research Council, P.le Fermi, 1-80055 Portici, Naples, Italy; martina.salzanodeluna@unina.it

**Keywords:** graphene, rubber, silica, wet compounding, latex mixing

## Abstract

The reduced graphene oxide (rGO) modified natural rubber composite (NR) filled with high contents of silica was prepared by a wet compounding and latex mixing process using a novel interface modifier cystamine dihydrochloride (CDHC) with coagulation ability. CDHC acts as a coagulation agent through electrostatic interaction with rGO, SiO_2_, and latex rubber particles during the latex-based preparation process, while in the obtained silica/graphene/natural rubber composites, CDHC acts as an interface modifier. Compared with the composites prepared by the conventional mechanical mixing method, the dispersion of both rGO and SiO_2_ in the composites made by a wet compounding and latex mixing process is improved. As a result, the obtained silica/graphene/natural rubber composite prepared by this new method has good comprehensive properties. A Dynamic Mechanical Test suggests that the tan δ values of the composites at 60 °C decrease, indicating a low rolling resistance with increasing the graphene content at a low strain, but it increases at a higher strain. This unique feature for this material provides an advantage in the rubber tire application.

## 1. Introduction

Inorganic fillers such as carbon black and silica (SiO_2_) are widely used in the rubber industry for various purposes, mainly for reinforcement, reduction in material costs, and improvements in processing. Compared with the most commonly used carbon black, silica is more helpful to increase the wet grip and reduce the rolling resistance of rubber materials. The latex compounding method, that is, mixing silica solution and rubber latex together first and then coagulating, is an innovative method to prepare the silica/rubber materials for tires [1,2,3,4,5]. However, due to the high polarity of the silica surface, it is difficult to disperse the silica particles in the non-polar rubber matrix, and the interfacial interaction between silica and rubber is not strong enough [6,7,8]. Generally, surface modification on silica is necessary in order to solve this issue [9,10,11,12].

Graphene possesses ultra-high mechanical strength, a high Young’s modulus, and high electrical and thermal conductivity, which outperform other materials [13,14,15,16,17]. Nowadays, graphene and other fillers such as carbon black [18,19], silica [20,21,22,23,24,25], ZnO [26], Al_2_O_3_ [27], Fe_3_O_4_ [16], carbon nanotubes [28], etc., was combined to enhance the property of rubber materials. For example, the silica/reduced graphene oxide (SiO_2_@rGO) hybrid fillers were prepared by solution electrostatic assembly, and the dispersion of graphene in the rubber matrix was improved [20,21,22]. This method can avoid the agglomeration of the graphene fillers; however, for high content SiO_2_, the aggregation of silica will occur, which may embed the graphene inside the agglomerates and restrict the reinforcing effect of graphene. Therefore, for high content silica graphene/rubber materials, which have real application in tires, it is still a big challenge to improve the dispersion of both graphene and silica fillers and attain strong interfacial interaction between fillers and rubber. 

The environment-friendly latex compounding method has been increasingly employed to prepare the rubber materials [29,30,31,32]. For the latex compounding method, the coagulation is the most critical step. Before coagulation, the fillers should be uniformly dispersed in the mixed latex, and the adsorption of filler on the surface of rubber latex particles should be strong to avoid the self-aggregation of the fillers and the pre-precipitation of fillers. During the coagulation process, for a stable mixed latex, the coagulation agent has to interact strongly with silica, graphene oxide, and latex particles, and thus allow the all components of the latex precipitate at the same time. 

**Figure 1:**
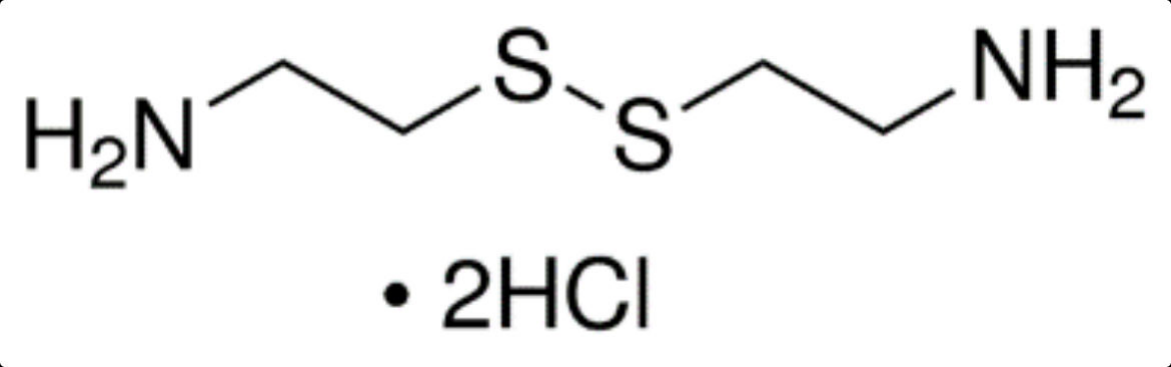
The chemical structure of cystamine dihydrochloride.

Here, the reduced graphene oxide modified natural rubber composite (NR) filled with high contents of silica (60 phr) was prepared by a wet compounding and latex mixing process using a novel interface modifier cystamine dihydrochloride (CDHC) with coagulation ability. CDHC with the amino group with positive charge in the both ends of the molecular chain (Figure 1) can act as the coagulation agent by electrostatic interaction. In addition, CDHC has a disulfide bond in the molecular structure, which will also participate in the vulcanization reaction of rubber and thus can improve the crosslinking efficiency of the rubber. For a comparative purpose, the SiO_2_/rGO/NR composites were also prepared by using the latex-based GE/rubber masterbatch and twin-roll mixing with silica (LMTM method). The effect of rGO on the mechanical property, dynamical property, water permeability, and electrical and dielectric properties were investigated. 

## 2. Materials and Methods

### 2.1. Materials

Natural rubber composite (NR) (SCR 5) was purchased from Yunnan Natural Rubber Industry Co, Ltd., Kunming, China, and the NR latex (solid content: 60 wt %) was provided by Chengdu Fangzheng Co., Ltd. (Chengdu, China). Graphene oxide was obtained from the Sixth Element Materials Technology Co., Ltd. (Changzhou, China). The purchased GO was washed several times until the pH was neutral. Nano-silica with an average size of 18–22 nm was provided by Nanjing Tianxing New Materials Company Co., Ltd. (Nanjing, China). Hydrazine hydrate and OP-10 were purchased from Chengdu Kelong Chemical Reagent Company (Chengdu, China). Cystamine dihydrochloride (CDHC) was purchased from Best Chemical Reagent Co., Ltd. (Chengdu, China). All the other reagents including sulfur, zinc oxide, N-cyclohexyl-2-benzothiazolesulfenamide (CBS), 2-mercaptobenzothiazole (MB), antioxidant (4010NA), and stearic acid were received and used without any purification.

### 2.2. Synthesis of SiO_2_/rGO/NR Composites

**Scheme 1:**
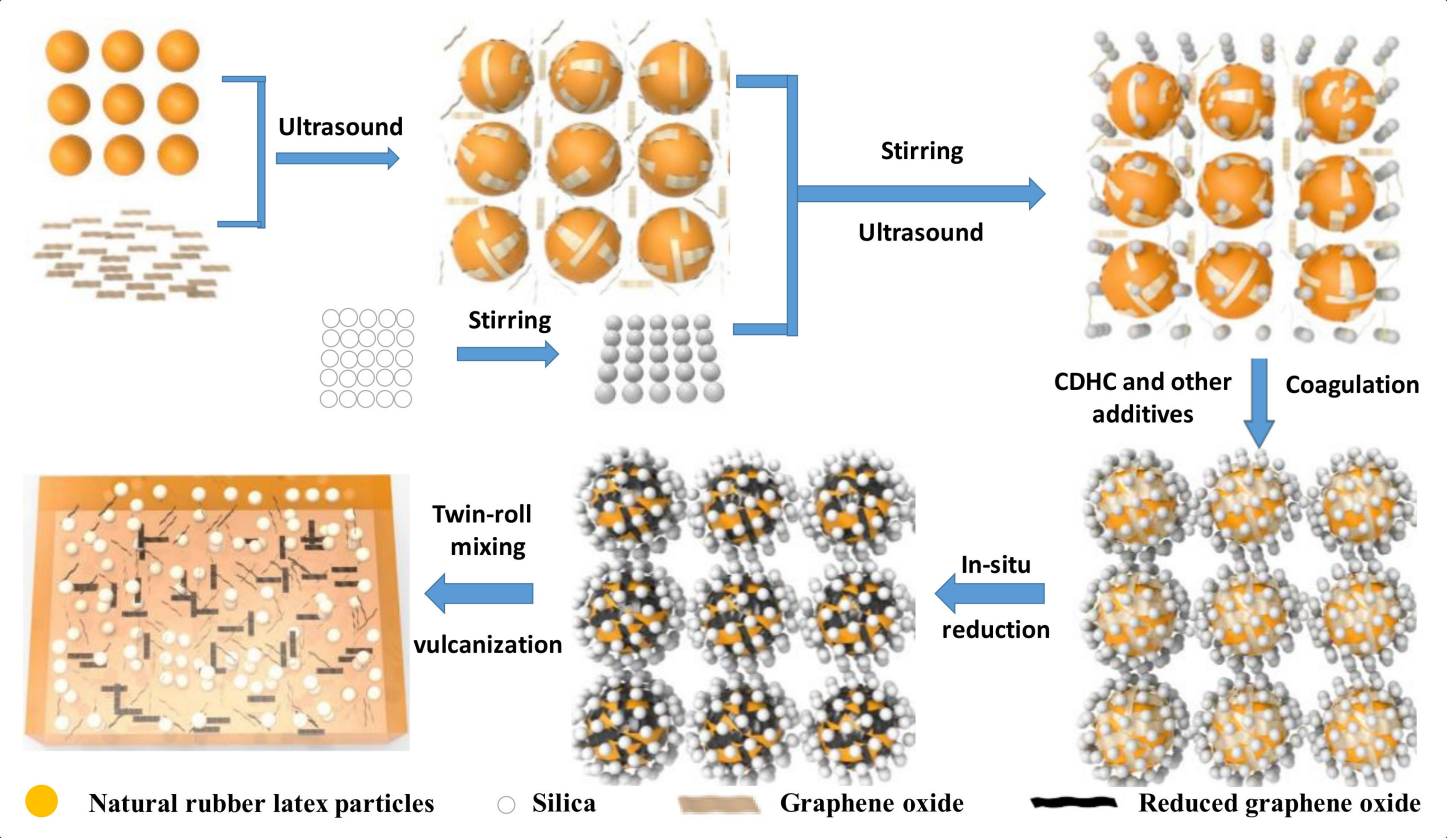
A wet compounding process combined with ultrasonically assisted latex mixing (WCL) method for the preparation of reduced graphene oxide (rGO)/silica/natural rubber (NR) composites.

Firstly, the nano-silica was re-dispersed in the deionized water (30 mg·mL^−1^) by ultrasonic and mechanical stirring for 5 h at room temperature simultaneously. A certain amount of GO was dispersed into deionized water by using an ultrasonic bath at 25 °C for 3 h to prepare 2 mg·mL^−1^ GO aqueous dispersion. Then, natural rubber latex (NRL) was mixed with the GO solution by sonication for 2 h. After that, the silica dispersion solution was added to the latex, and then the latex mixture was agitated by mechanical and ultrasonic mixing for 2 h. Then, the other vulcanization additives with surfactant OP-10 (the formula is given in Table 1), which were pre-emulsified by a high-speed mixer (ULTRA-TURRAX IKA T25, Staufen, Germany), were added and dispersed in latex mixture by ultrasound and stirring for 2 h. Then, the CDHC solution was dropped into the latex for coagulation. Then, hydrazine hydrate was added to the system under ultrasonic and mechanical stirring for 2 h at 60 °C, in order to reduce GO to rGO in the system. The resulting coagulated SiO_2_/rGO/NR solid was filtered and washed with water several times and vacuum-dried in an oven at 60 °C for 30 h. The dried SiO_2_/rGO/ NR solid was mixed for 5 min in an open twin-roll mill (LRM-S-150, Labtech, Samutprakarn, Thailand) at room temperature with a friction ratio of 1:1.1 and a nip gap of about 0.5mm. The mixed pastes were compressively molded at a temperature of 150 °C and a pressure of 15 MPa for the optimum curing time, and they were cooled under a pressure of 10 MPa to room temperature for 3 min to get the different vulcanized samples. The preparation process is shown in Scheme 1. 

The control samples made by the LMTM method were prepared as follows: The rGO/NR masterbatch with 10 wt % rGO was prepared by an ultrasonically assisted Latex Compounding Method [29]. In detail, natural rubber latex (NRL) was mixed with the GO solution by sonication, and hydrazine hydrate was used to reduce the GO. Then, CDHC was added to coagulate this latex, which after filtration was washed and vacuum-dried. The obtained rGO/NR materbatch and silica powder were mixed with commercial NR in a Banbury mixer. Finally, the other vulcanization additives in Table 1 were mixed with the composite rubber by twin roll mill, and the resulting composites were cured at 150 °C to get the crosslinked samples. The obtained composites were designated as W-x and L-x, in which x represents the rGO content, W represents the composites made by the wet compounding process combined with ultrasonically assisted latex mixing (WCL), and L represents the composites made by the LMTM method. For example, W-0.5 represents the 60SiO_2_/0.5rGO/NR composites with 0.5 phr rGO and 60 phr silica.

### 2.3. Characterization

Zeta potential was measured by using a Malvern Zetasizer Nano-ZS (Malvern, UK). All solutions were diluted to 300 μg·mL^−1^. All the samples were tested 3 times, and average values were recorded. 

XPS measurements of the composites were carried out by using an Kratos XSAM 800 (Kyoto, Japan) in the analysis chamber under a pressure of 2 × 10^−7^ Pa. 

The crosslink density could be expressed by Flory–Rehner equation [33].

(1) $$v_{e}=\frac{\rho_{d}}{M_{C}}=-\frac{ln(1-v_{r})+v_{r}+\chi v_{r}{}^{2}}{v_{1}\left(v_{r}{}^{\frac{1}{3}}-\frac{v_{r}}{2}\right)}$$

where *ν_e_* is the network chain density (mol/cm^3^), *v_r_* is the volume fraction of rubber in the swollen network, *ν_1_* is the molar volume of toluene (106.3 cm^3^/mol), and *χ_1_* is the Flory/Huggins interaction parameter between toluene and rubber (0.391). 

The bound rubber content was evaluated by using the method reported by Leblanc and Hardy [34]. An uncured sample (0.5 g) was cut into small pieces and placed into a steel wire net (*m_1_*). The net was closed to form a sort of basket, weighed (*m_2_*), and then immersed in toluene at room temperature for 72 h during which the sample was washed with fresh solvent every 24 h. The basket was slowly removed from the solvent and dried at room temperature under vacuum for a few hours until a constant weight (*m_3_*) was achieved. The amount of bound rubber (as the weight percent, wt %) of the initial rubber content of the uncured sample was given by Equations (2) and (3):

(2) $$BdR\left(\%\right)=\frac{m_{0}-\left(m_{2}-m_{3}\right)}{m_{0}}\times 100$$

(3) $$m_{0}=\left(m_{2}-m_{1}\right)\times \varphi$$

where *m_0_* is the rubber content in the sample, *m_1_* is the mass of the empty net, *φ* is the mass fraction of the rubber in the composites, *m_2_* is the mass of the basket with the unextracted sample, and *m_3_* is the mass of the basket with the extracted dried sample.

Transmission Electron Microscopy (TEM) analysis was performed by using an FEI TecnaiG_2_ F_20_ S-TWIN (Hillsboro, OR, USA) transmission electron microscope, operating at an accelerating voltage of 200 kV. The measurement of mechanical properties was conducted on a universal testing machine (Instron 5567, Boston, MA, USA) at room temperature.

For tensile tests, the dumbbell-shaped specimens (75 mm in length, 4 mm in width, and 2 mm in thickness) were stretched until break at a cross-head rate of 500 mm/min. The stress/strain curves were recorded. The tensile strength, elongation at break, and stress at 100%, 200%, and 300% strain were measured with the average of seven specimens taken as the final value. The previous test shows that there is no difference between NR and NR latex, so it is significant to compare the composites that were made by the WCL and LMTM method [35]. 

Dynamic mechanical tests were performed by using a TA Q800 (New Castle, DE, USA) DMA instrument at a frequency of 10 Hz in a tensile mode. The values of tan δ were evaluated at a constant strain of 0.1% from −90 to 80 °C at a heating rate of 3 °C /min. Then, the values of tan δ were evaluated at a constant temperature of 60 °C with a strain sweep of 0.01–40% at a static preload of 0.01 N.

The measurements of water vapor permeability were performed by using the infrared sensor technique by means of a PermatranW3/31 (Mocon, Minneapolis, MN, USA). The samples with a surface area of 5 cm^2^ and 300 μm thickness were tested at 25 °C and 50% Relative Humidity (RH). 

The electrical conductivity of the composites was determined by a two-probe method with a picoammeter (Keithley 2400, Keithley Instruments Inc, Solon, OH, USA). The measured volume resistance (Rv) was converted to volume conductivity (σ) using Equation (4):

(4) $$\sigma =\frac{d}{RvS}$$

where *d* is the distance between two measuring electrodes (m), *Rv* is the measured resistance (*Ω*), and *S* is the effective area of the measuring electrode. The specimens were coated with a silver paste before the test. 

The dielectric properties were performed using a rotational rheometer (ARES, Rheometrics Scientific, New Castle, DE, USA) equipped with a dielectric thermal analysis tool, constituted by a couple of stainless steel parallel plates (diameter 25 mm) connected with an LCR Meter (E4980A, Agilent, Santa Clara, CA, USA). Measurements were carried out on films of about 100 mm in thickness. The dielectric response was measured at room temperature by monitoring the real parts of the complex permittivity as a function (*ε′*) of the frequency of the alternating electrical field, 10^2^–10^6^ Hz. The dielectric constant, *ε_c_*, can be estimated by using Equation (5)

(5) $$\varepsilon_{c}=\frac{\varepsilon^{\prime }}{\varepsilon_{0}}$$

where *ε_0_* =8.85 × 10^−12^ Fm^−1^ is the permittivity of free space.

The NRL contains 60 wt % solid NR content. All the samples contain the same process additives (sulfur 2 phr, zinc oxide 5 phr, CBS 1.5 phr, antioxidant 4010NA 1 phr, and stearic acid 2 phr).

## 3. Results and Discussion

### 3.1. Effect of CDHC on Preparation of SiO_2_/rGO/NR Composites

**Figure 2:**
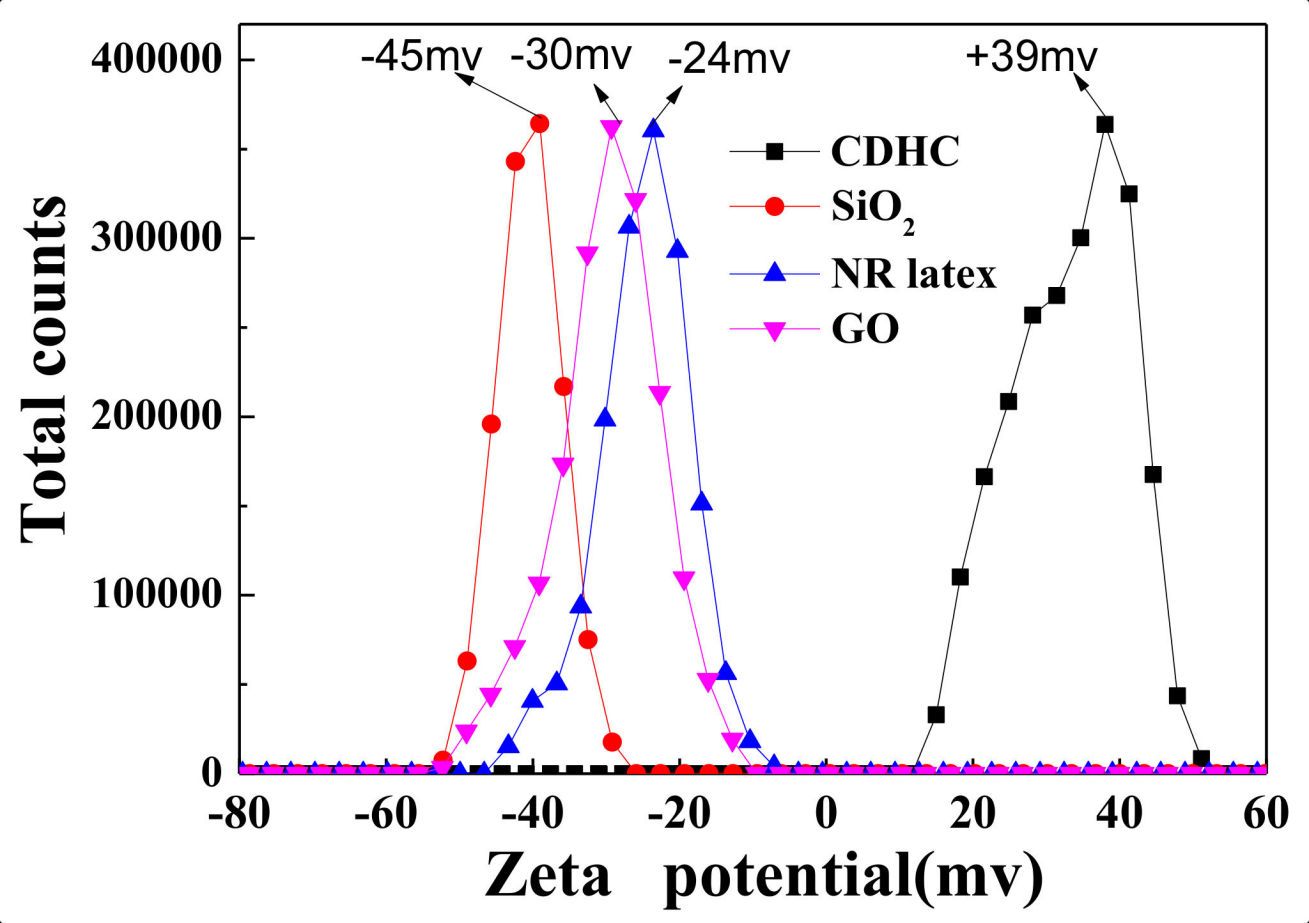
The zeta potential of GO, silica, NR latex, and cystamine dihydrochloride (CDHC) solution.

**Scheme 2:**
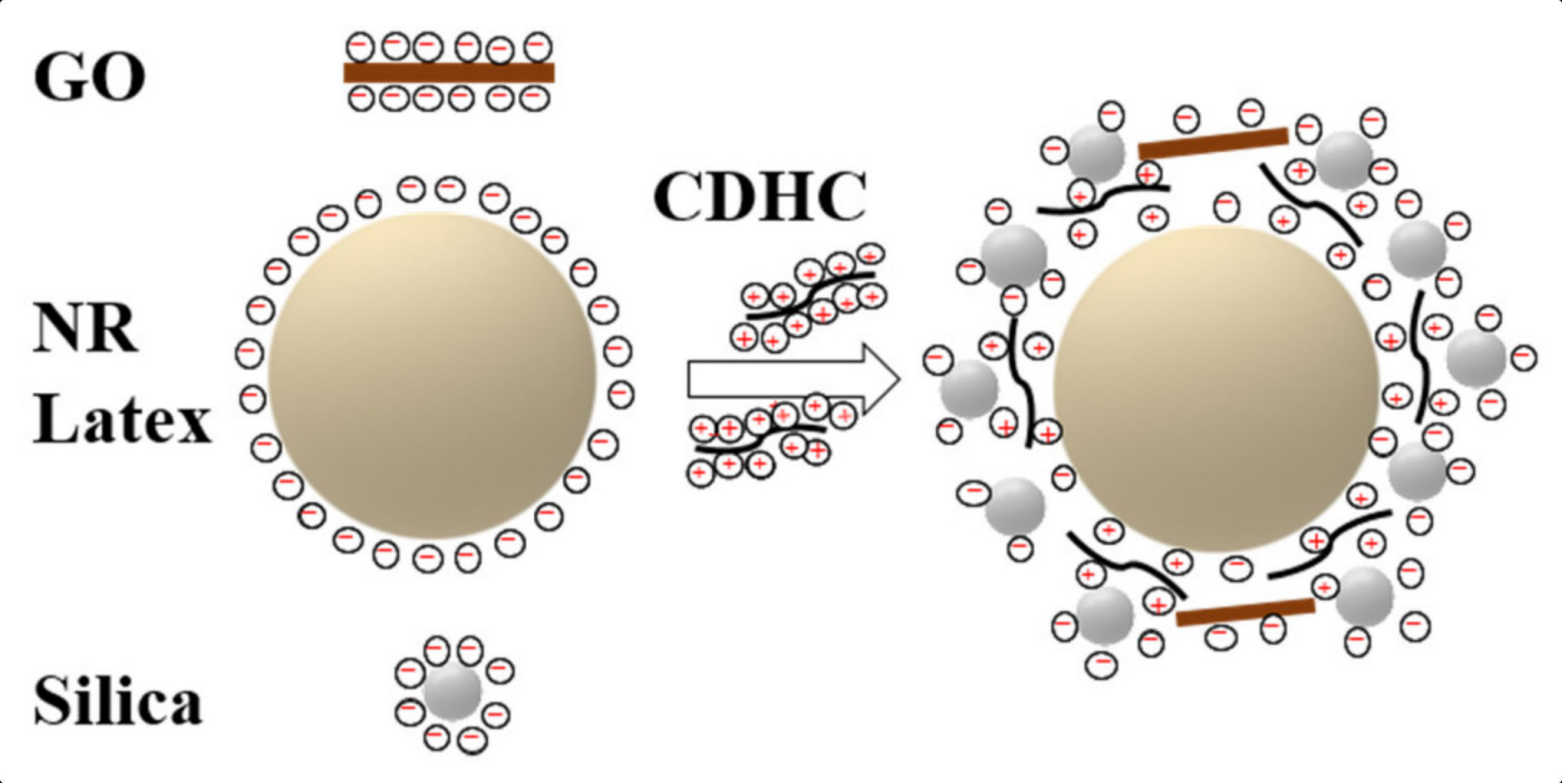
The coagulation mechanism of CDHC.

Zeta potential allows evaluating the stability of colloid and the interaction force between colloid and solute additives. Figure 2 shows the zeta potential for the fillers and CDHC used for the preparation of rubber composites with GO. The results show that silica and NR latex are negatively charged, while the CDHC solution is positively charged. Therefore, CDHC can form strong electrostatic interactions with rGO, silica, and NR latex particles, which will destroy the stability of latex and cause the coagulation of all components. The coagulation mechanism of CDHC is shown in Scheme 2.

**Figure 3:**
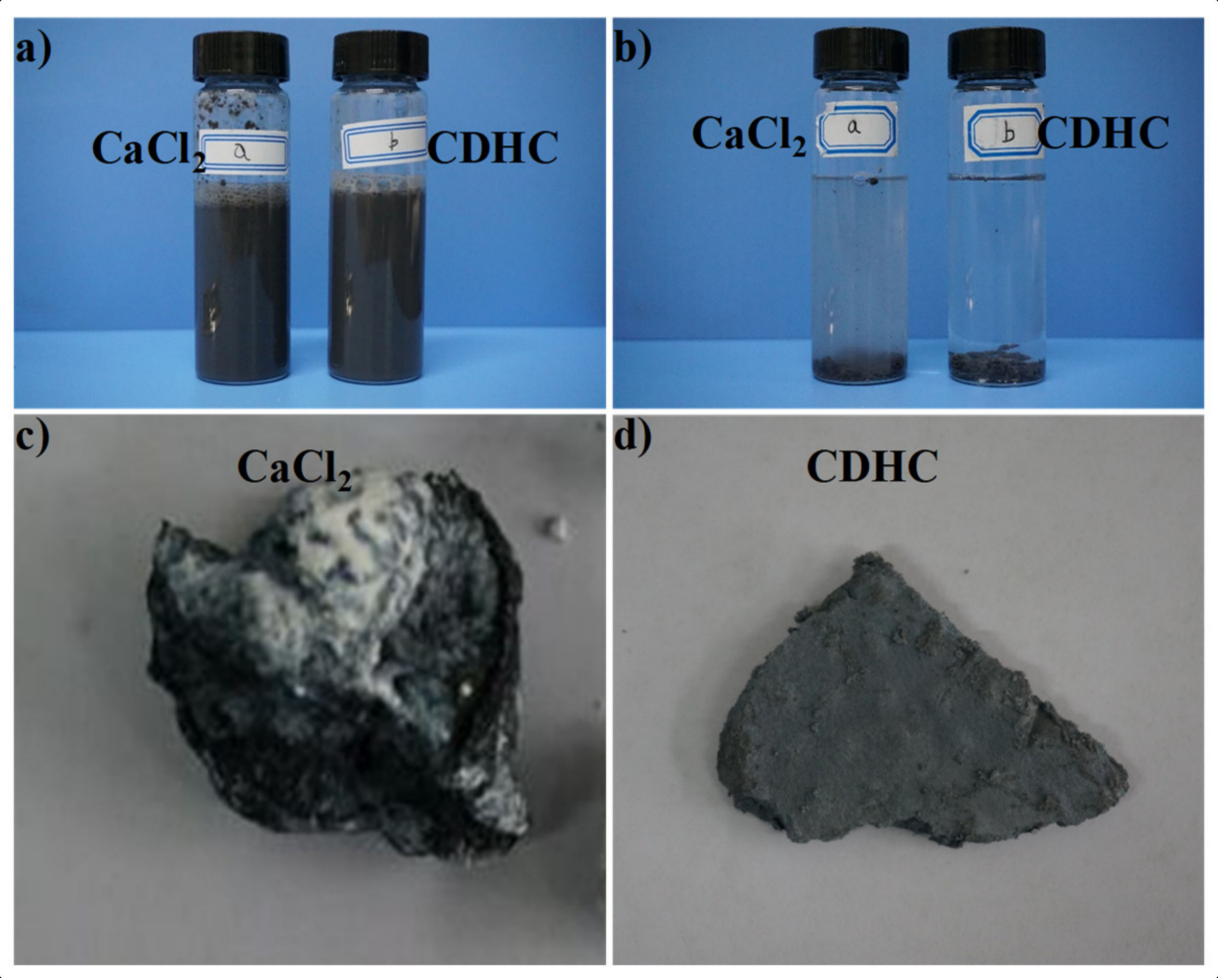
The difference of the coagulation effect of CaCl_2_ (left) and CDHC (right) on the rGO/silica/NR latex mixture in different periods (**a**) adding coagulation agents; (**b**) the coagulated and filtered sample was re-added into water; (**c**) the CaCl_2_ coagulated sample; (**d**) the CDHC coagulated sample.

Figure 3 shows the coagulation effect of CDHC and CaCl_2_ on an SiO_2_/rGO/NR latex mixture. From Figure 3a, we can see that when the CDHC or CaCl_2_ solutions were dropped into the rGO/silica/NR latex mixture, both systems were coagulated into the small particles, and there was no significant difference between the two coagulation agents. Then, after the mixed latex was flocculated for a while, the coagulated samples were obtained by filtration and washing and then added into water by sonication. It was found for a CaCl_2_ coagulated sample that the supernatant is a little turbid (Figure 3b), while for the CDHC coagulated sample, it is completely transparent. This phenomenon shows that some silica in the CaCl_2_ coagulated sample was re-entered into the aqueous solution, indicating that the silica was not well attached with NR latex. On the other hand, the silica in the CDHC coagulated sample has a good interaction with the NR latex and could not be re-entered into the aqueous solution. Figure 3c shows the surface appearance for the dried coagulates. Some white powders appeared on the surface of the CaCl_2_ coagulated sample, while for the CDHC coagulated sample, no white powders are observed, which indicates a uniform coagulation. All the results confirm that the CDHC has a better coagulation effect due to a strong interaction between silica and the NR matrix, which is beneficial to the enhancement of the composite’s properties.

**Figure 4:**
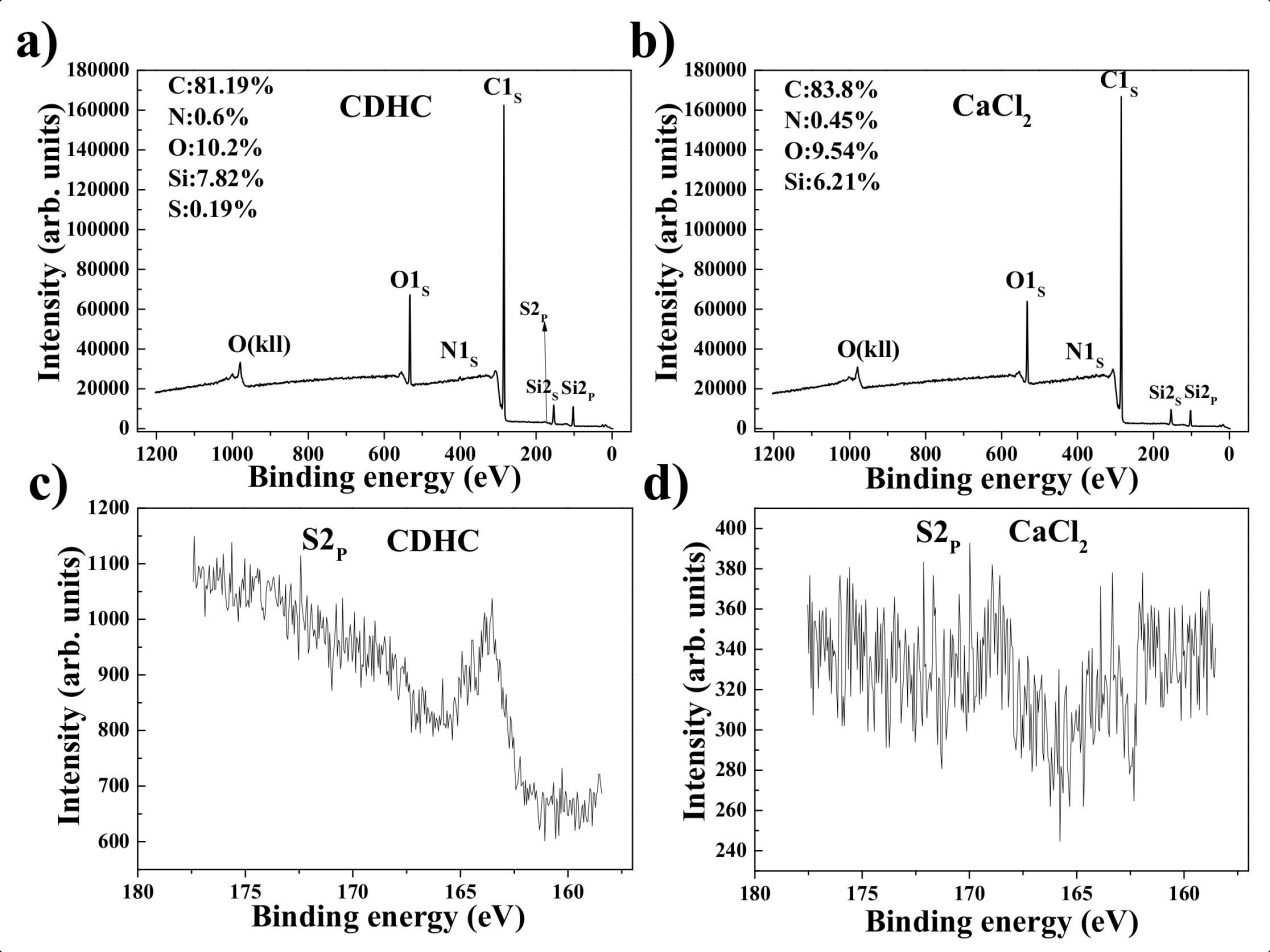
XPS wide scan and carbon narrow scan of the composites which coagulated by (**a**), (**c**) CDHC and (**b**), (**d**) CaCl_2_.

XPS tests were used to compare the coagulation effect of CDHC and CaCl_2_ on the preparation of SiO_2_/rGO/NR composites. The samples used in the tests did not contain any vulcanization additives. Figure 4 shows the XPS of composites which were coagulated by the CDHC and CaCl_2_. The coagulated sample has higher N, S, Si, and O weight content compared with the sample coagulated by CaCl_2_. This confirms that after several washings, as described in the preparation procedure, the water-soluble CDHC (which has the N, S element on its molecules) also existed in the composites. The sample coagulated by CaCl_2_ exhibits a lower Si and O weight content, indicating that some SiO_2_ particles in the latex mixture did not enter into the coagulation composites and have been washed away during the washing process. Above all, the CDHC acts as an intermediate substance able to “bind” rubber matrix, silica, and graphene well, which indicates that CDHC can act as the coupling agent that produces a stronger interfacial interaction between the fillers and rubber. 

**Figure 5:**
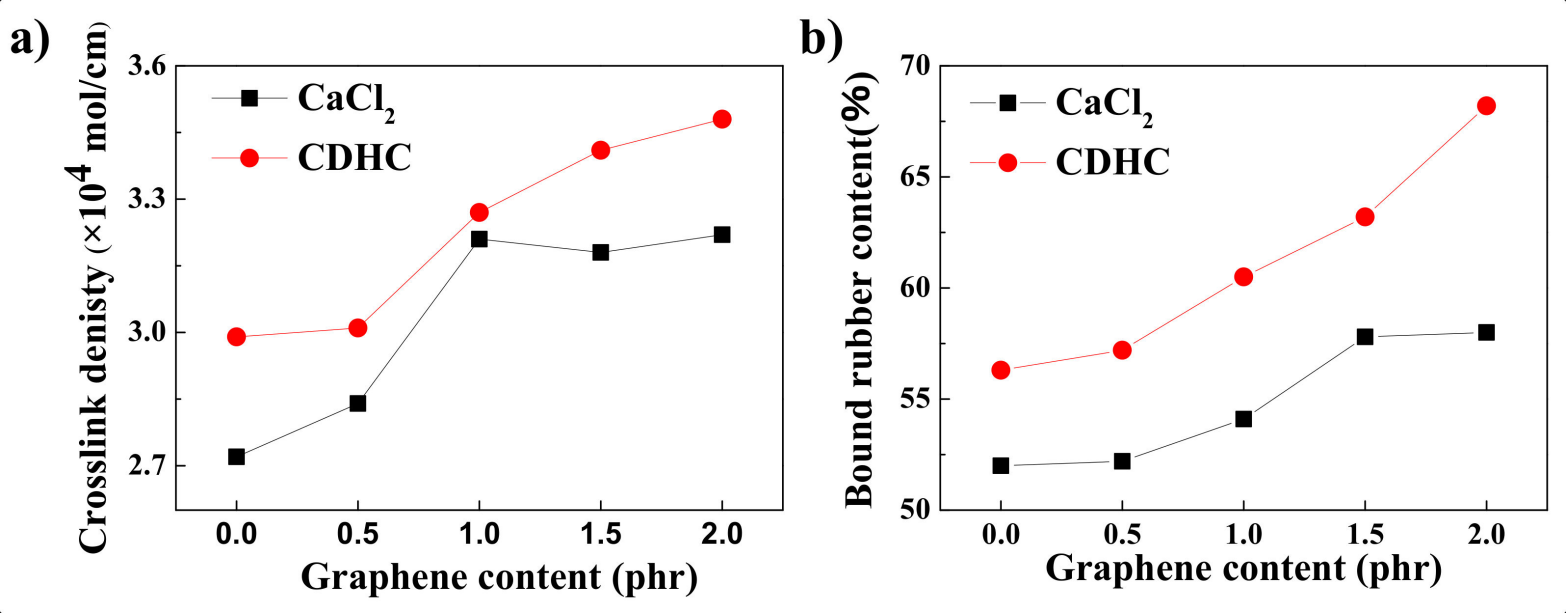
(**a**) Crosslinking density and (**b**) bound rubber content of different 60SiO_2_/rGO/NR composites, which were coagulated by CaCl_2_ and CDHC.

The crosslinking density and bound rubber contents were measured for the rGO/silica/NR nanocomposites which coagulated by CDHC and CaCl_2_ to investigate the interaction of graphene and silica with the rubber matrix. Figure 5 shows that the crosslinking density and the bound rubber content increase with the increasing amount of rGO. The larger surface area of rGO (compared to silica) leads to more physical crosslinking points between the filler and rubber matrix. Compared with the CaCl_2_ coagulated samples, the CDHC coagulated composites have a higher crosslink density and bound rubber content at the same rGO content. This is because the addition of CDHC containing sulfur in the molecular structure can promote the vulcanization to increase the crosslink density. In fact, during the preparation, the disulfide bond is reduced to a thiol bond and will generate sulfur free radicals during the vulcanization process. In the following study, for all the SiO_2_/rGO/NR composites samples obtained by the WCL method, CDHC was used as a coagulation agent unless specifically stated. 

### 3.2. Dispersion of Graphene and Silica in SiO_2_/rGO/NR Composites

**Figure 6:**
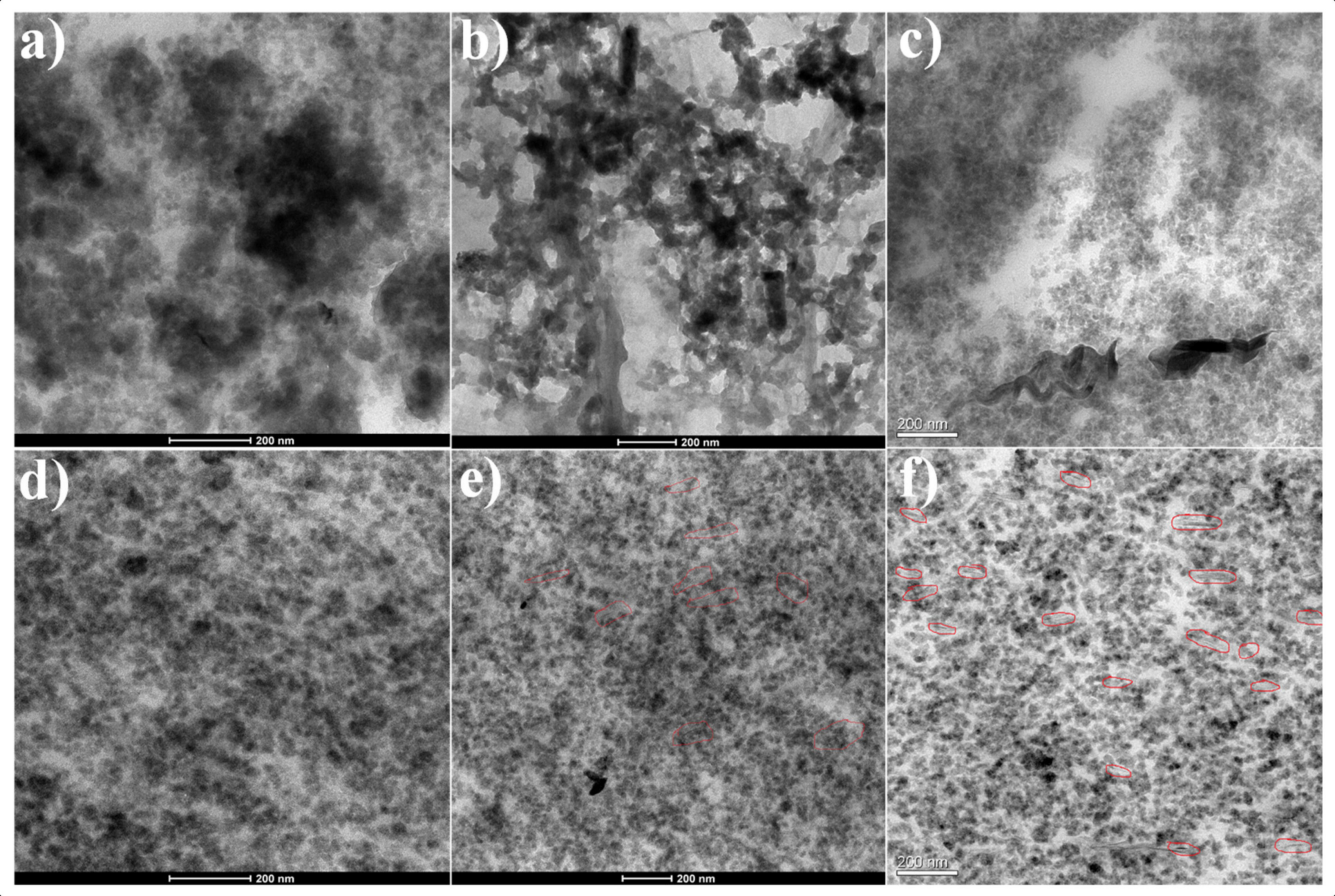
TEM images of the 60SiO_2_/NR composites prepared by (**a**) the rubber masterbatch and twin-roll mixing with silica (LMTM) method and (**d**) WCL method; TEM images of the 60SiO_2_/1rGO/NR composites prepared by (**b**) the LMTM method and (**e**) WCL method; TEM images of the 60SiO_2_/2rGO/NR composites prepared by (**c**) the LMTM method and (**f**) WCL method.

The dispersion morphologies of rGO and silica in the rubber matrix were investigated by TEM, and the images are shown in Figure 6. The long strip shapes represent the rGO nanoplatelets, and the small spherical particles represent the silica particles. Two methods, i.e., WCL and LMTM, were compared. As shown in Figure 6a–c, for LMTM, some silica aggregates can be observed, and some graphene is possibly embedded into the silica aggregates and hard to be distinguished in Figure 6b. This is due to the high viscosity of the graphene/rubber masterbatch and the poor interaction between the silica and rubber. However, Figure 6d–f suggest that for the WCL method, a good dispersion of both silica and graphene can be obtained at the nanoscale. Even in the high silica content composite, the rGO can be well dispersed in the rubber matrix. It can observed that the rGO is preferably distributed in the rubber matrix rather than embedded in the silica particles. This is very important, because graphene may play its role on the nanoscale by connecting with NR macromolecular chains. If graphene is embedded in the silica aggregates, it may not have a reinforcing effect, because it cannot contact with the rubber molecular chain. We believe that only if rGO is individually dispersed within the polymer matrix similar to a giant molecule and forms a strong combination with the polymer chain (possibly chemically bonded) can the nano-effect of graphene be realized. In addition, the role of silica should not be ignored, and its better dispersion can surely maximize the reinforcing effect for a rubber matrix. 

### 3.3. Mechanical Properties of Different 60SiO_2_/rGO/NR Composites

**Figure 7:**
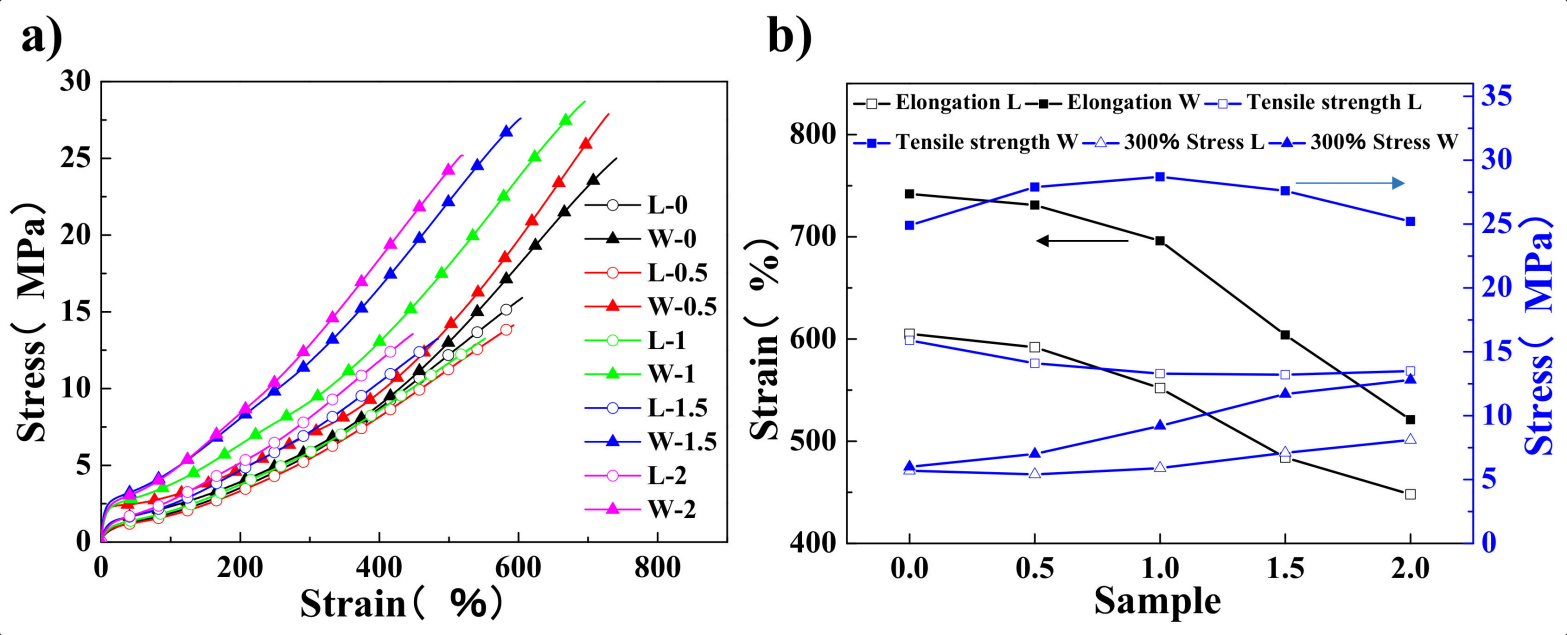
(**a**) The stress–strain curves of the rGO/60SiO_2_/NR composites with different rGO contents, prepared by two different methods. (**b**) The elongation (black line), tensile strength (blue line), and 300% stress (blue line) of different rGO/60SiO_2_/NR composites made by the WCL (solid point) and LMTM (hollow point) method.

Figure 7 shows the mechanical properties of composites prepared by using the two methods, i.e., LMTM and WCL. Both WCL and LMTM methods contribute to improving the tensile strength and modulus of the composites with the addition of rGO. However, the mechanical properties including the tensile strength, elongation at break, and stress at 300% elongation of the composites made by the WCL method are much better than those of composites made by the LMTM method. For example, for 1rGO/60SiO_2_/NR composites, the tensile strength of the sample prepared by the WCL method is 117% higher than that of the LMTM method. The tensile strength of 60SiO_2_/NR composites without graphene made by the WCL method is equal to 25 MPa, which is similar as previously reported using different processing [2,36]. For the rGO/SiO_2_/NR composites made by the LMTM method, the addition of rGO decreases the tensile strength, elongation break, and slightly increases the stress at 300% elongation. This is possibly because rGO is difficult to be well dispersed in the high silica content rubber matrix by twin-roll mixing. The addition of 0.5rGO into the 60SiO_2_/NR system made by the WCL method increases the tensile strength, and modulus, while the elongation break is nearly kept the same. An appropriate amount of rGO can be dispersed well in the rubber/silica matrix, resulting in stronger interfacial interactions, more efficient energy dissipation, and load transfer. When the amount of rGO increased up to 1 wt %, the tensile strength was enhanced to 28.7 MPa, but the elongation decreased slightly. With further increasing the amount of rGO, the tensile strength and elongation decreased, but the stress of 300% elongation increased. These results also indicate that the CDHC coagulation is feasible for the rGO/SiO_2_/NR composites.

### 3.4. Dynamic Mechanical Properties of 60SiO_2_/rGO/NR Composite

**Figure 8:**
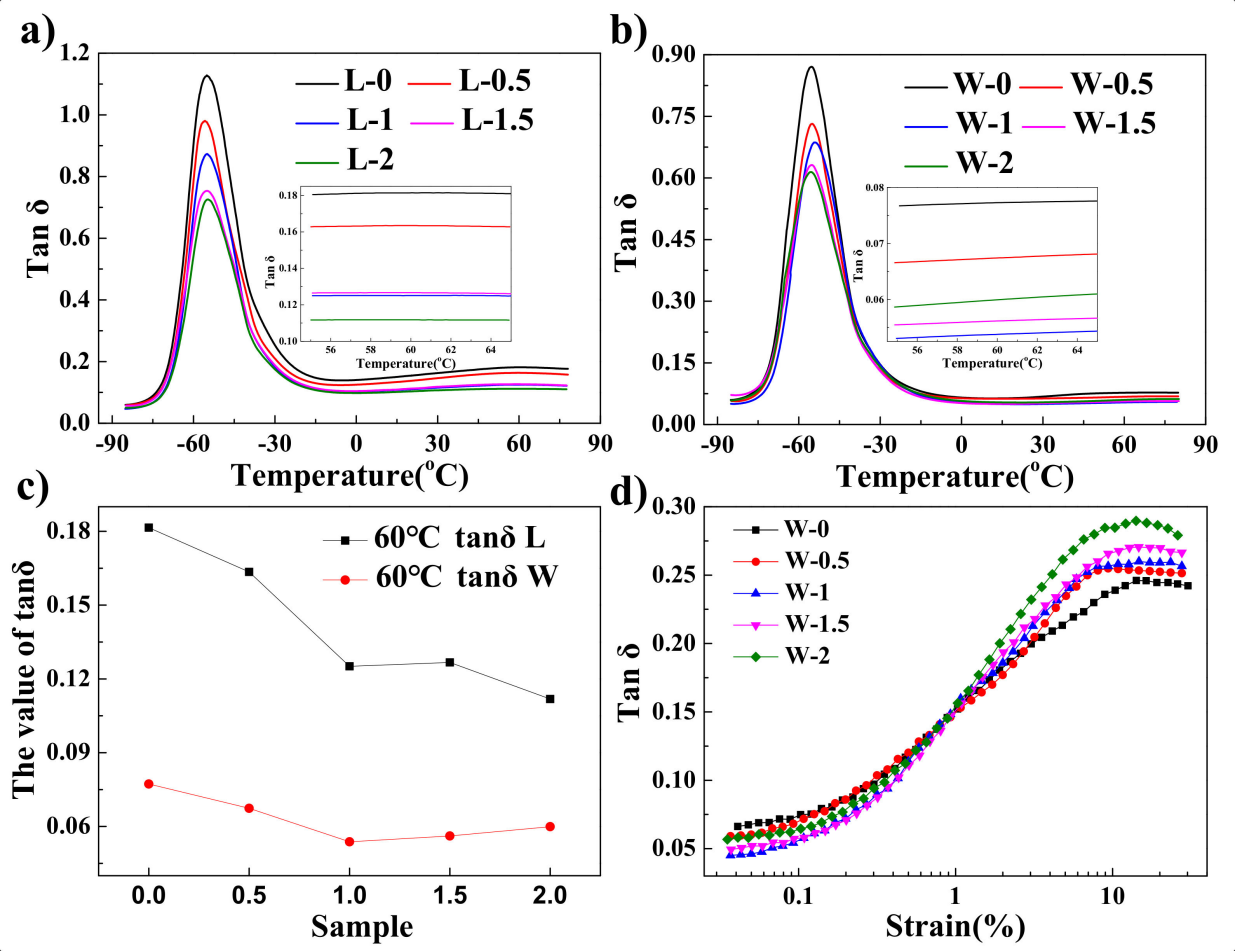
The dynamic properties of the different rGO/60SiO_2_/NR composites with different rGO contents. (**a**) Loss factor of the composites made by the LMTM method. (**b**) Loss factor of the composites made by the WCL method. (**c**) Loss factor of different composites at 60 °C made by the WCL method and LMCM method. (**d**) Loss factor of the composites made by the WCL method with different strains at 60 °C.

The dynamic mechanical properties of the 60SiO_2_/rGO/NR composites are shown in Figure 8. The values of tan δ were evaluated by the constant strain mode, and the constant temperature mode for the WCL and LMTM composites are shown in Figure 8a,b, respectively. The addition of rGO causes the reduction of maximum values of tan δ of the composites in both WCL and LMTM methods. This is attributed to the physical and chemical adsorption of interaction on the rubber chain on the surface of the filler, which reduces the chain mobility [37,38]. The Tg of the composites increases slightly with the addition of rGO into the rubber matrix made by WCL method. This suggests that the graphene can disperse well in the silica/rubber matrix made by the WCL method. After adding rGO, the Tg of the composites prepared by the WCL method increases slightly, indicating that graphene has a certain interaction with the rubber molecular chain, which is the result of good dispersion of graphene in the rubber matrix. 

For a rubber-filler system, there is an accepted rule of thumb, i.e., a lower value of tan δ at 60 °C suggests a lower rolling resistance [20,25]. The tan δ values of the composites at 60 °C prepared by the WCL method are much lower (almost decreases of 50%) than those of composites prepared by the LMTM method with the same graphene content, indicating a lower rolling resistance (see Figure 8c). The better dispersion of rGO and silica in the rubber matrix likely allows energy to dissipate more efficiently, which will help reduce internal friction. Furthermore, the introduction of rGO in the composites prepared by both the WCL and LMTM methods plays a key role in decreasing the rolling resistance. This is because the rGO has a stronger affinity with the rubber matrix, which enhances the filler–matrix interaction and decreases the internal friction. A further addition of rGO (> 1 wt %) leads to a slight increase in the rolling resistance of the composites prepared by the WCL method, because the aggregation of rGO results in a non-uniform stress distribution. 

It can be seen from Figure 8d that values of tan δ at 60 °C increase sharply with the increase of the strain, and the values of tan δ tend to be stable after strain reaches 10%. At higher strains, the graphene-containing composites have higher tan δ values than the graphene-free ones, which indicates that graphene will increase the rolling resistance in a higher stretching stain condition. At a strain of 15%, the tan δ value for the sample at 2% graphene content (W-2) improved by ≈15% compared to the sample without graphene (W-0). This unique feature may contribute to shortening the braking distance when this rubber material is used for automobile or bicycle tires. 

### 3.5. Water Vapor Permeability Properties

**Figure 9:**
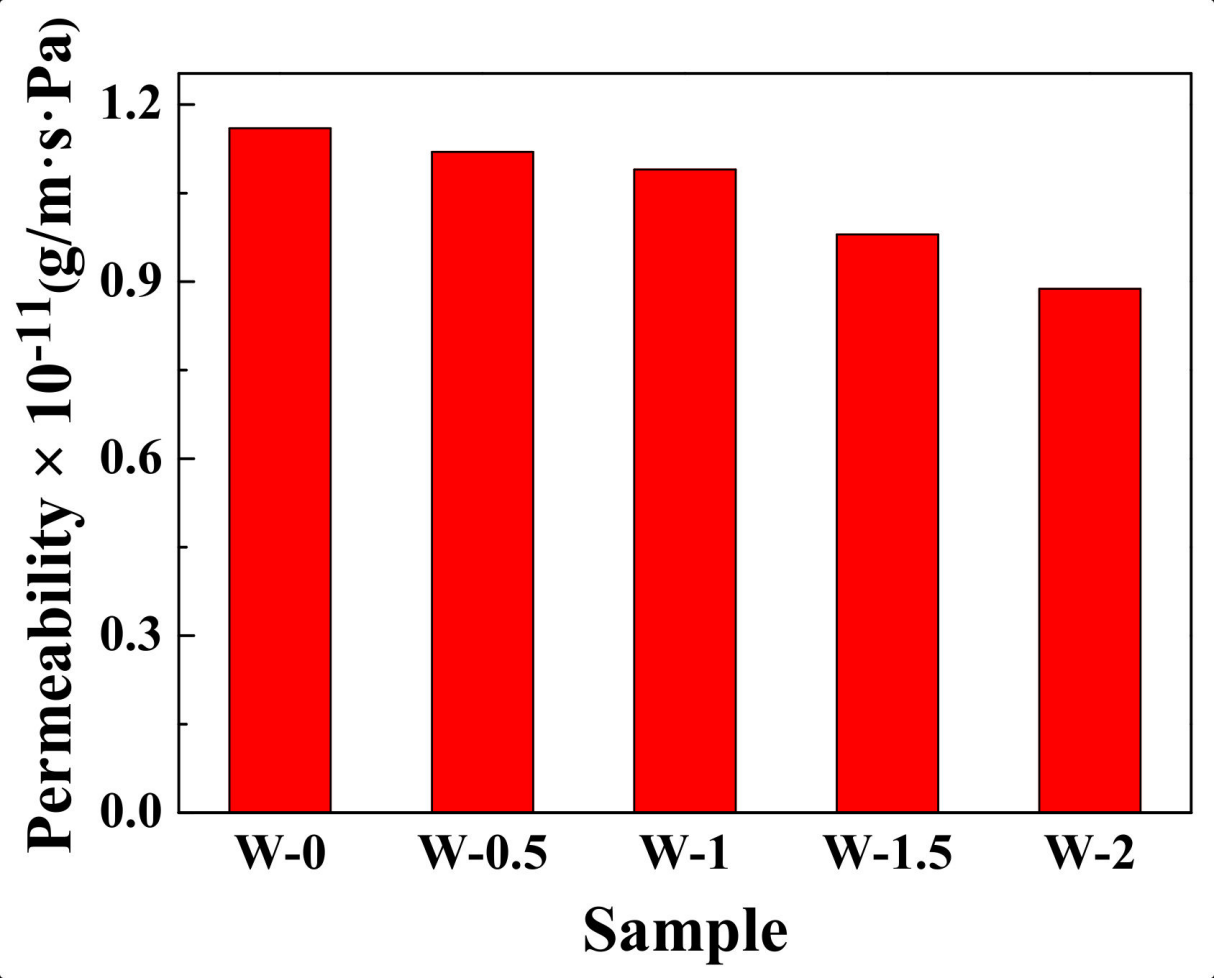
Water vapor permeability of 60SiO_2_/rGO/NR composites with different rGO contents.

Water vapor permeability data for composites prepared by the WCL method are shown in Figure 9**.** The water permeability of the rubber composites decreased by increasing the rGO content. When the graphene content reaches 2 wt %, the water permeability decreases by ≈26.1% compared with the composites without graphene. The presence of graphene will increase the diffusion distance and contribute to reduce the free volume of rubber, resulting in enhanced water barrier properties [39,40]. The improvement of the barrier properties of the rubber-based composites is important for expanding the range of applications such as a seal and inner tire. 

### 3.6. Dielectric and Electrical Property

**Figure 10:**
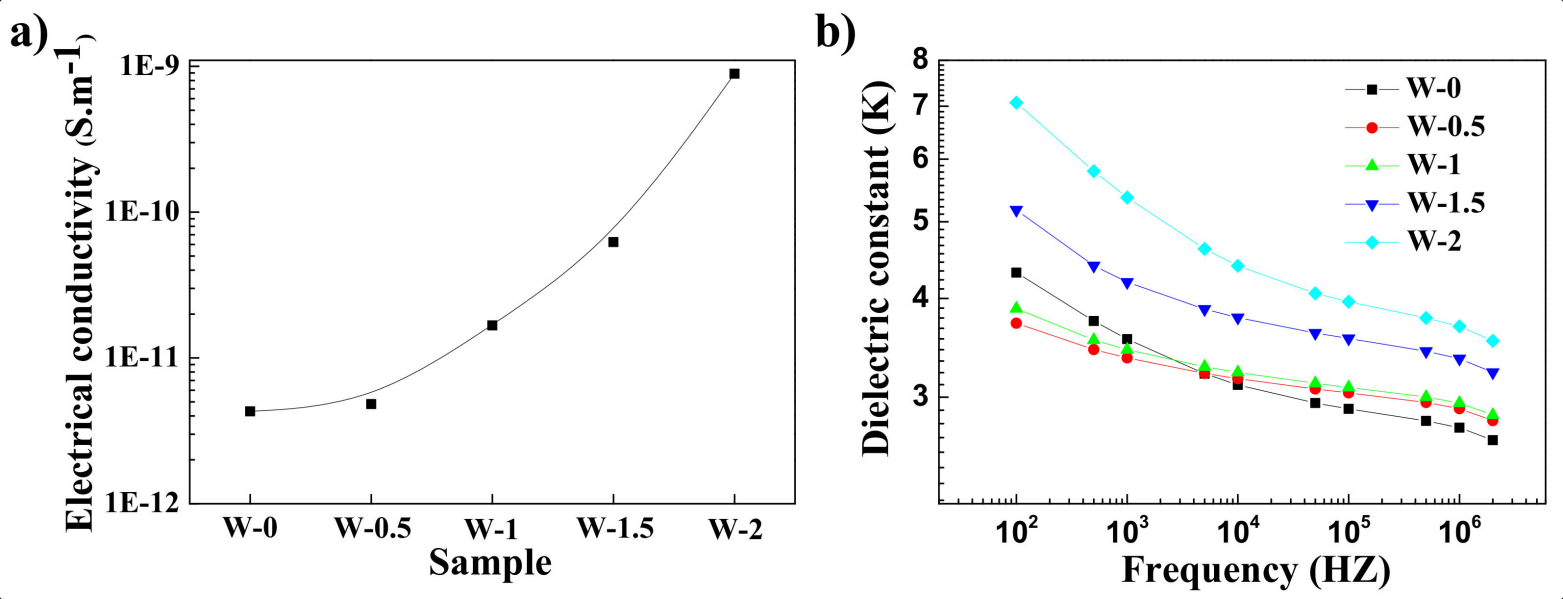
(**a**) Electrical conductivity and (**b**) dielectrical constant of rGO/60SiO_2_/NR composites made by the WCL method.

Figure 10 shows the electrical and dielectric properties of the several composites. Compared to the rGO/NR composites without silica content [29], the electrical conductivity for SiO_2_/rGO/NR decreases. The high SiO_2_ content may break the graphene conductive contact and network. The conductivity of the composites increases with the increase of rGO content. The dielectric constant also increases with the enhanced rGO content, especially in the lower frequency (see Figure 10b). In particular, the dielectric constant increases from 3.7k to 7.1k as the rGO content increases from 0 to 2 phr. This is because conductive graphene can improve the capacity of the charge storage at the internal interfaces between fillers and rubber [32,41].

## 4. Conclusions

A modified latex process, i.e., a wet compounding process combined with ultrasonically assisted latex mixing (WCL), was used to prepare the high silica content rGO/NR composites with the assistance of cystamine dihydrochloride (CDHC) as a multifunctional modifier. CDHC can form strong electrostatic interactions with rGO, silica, and NR latex particles, and thus act as a coagulation agent during the preparation process and also the interface compatibilizer in the obtained composites. CDHC also promotes the vulcanization confirmed by the bound rubber content and crosslink density. TEM shows that both graphene and SiO_2_ are well dispersed in natural rubber by this method. The obtained silica/graphene/natural rubber composite prepared by this new method exhibits good mechanical properties and low water vapor permeability. A DMA Test suggests that the tan δ values of the composites at 60 °C decreased with increasing the graphene content at a low strain, but increased at a higher strain, which provides a unique advantage for this material in the rubber tire application. 

## Figures and Tables

**Table 1:** Formulations of rGO/silica/NR composites.

Sample	rGO	Silica	CDHC	NRL	Raw NR
60SiO_2_/NR (W-0)	0	60	10	166.7	0
60SiO_2_/0.5rGO/NR (W-0.5)	0.5	60	10	166.7	0
60SiO_2_/1rGO/NR (W-1)	1	60	10	166.7	0
60SiO_2_/1.5rGO/NR (W-1.5)	1.5	60	10	166.7	0
60SiO_2_/2rGO/NR (W-2)	2	60	10	166.7	0
60SiO_2_/NR (L-0)	0	60	10	0	100
60SiO_2_/0.5rGO/NR (L-0.5)	0.5	60	10	7.5	95.5
60SiO_2_/1rGO/NR (L-1)	1	60	10	15	91
60SiO_2_/1.5rGO/NR (L-1.5)	1.5	60	10	22.5	86.5
60SiO_2_/2rGO/NR (L-2)	2	60	10	30	82
